# Failure to see money on a tree: inattentional blindness for objects that guided behavior

**DOI:** 10.3389/fpsyg.2014.00356

**Published:** 2014-04-23

**Authors:** Ira E. Hyman, Benjamin A. Sarb, Breanne M. Wise-Swanson

**Affiliations:** Department of Psychology, Western Washington UniversityBellingham, WA, USA

**Keywords:** inattentional blindness, cell phone, attention, visual attention, perception-action dissociation, ventral and dorsal visual streams, two visual systems hypothesis

## Abstract

How is it possible to drive home and have no awareness of the trip? We documented a new form of inattentional blindness in which people fail to become aware of obstacles that had guided their behavior. In our first study, we found that people talking on cell phones while walking waited longer to avoid an obstacle and were less likely to be aware that they had avoided an obstacle than other individual walkers. In our second study, cell phone talkers and texters were less likely to show awareness of money on a tree over the pathway they were traversing. Nonetheless, they managed to avoid walking into the money tree. Perceptual information may be processed in two distinct pathways – one guiding behavior and the other leading to awareness. We observed that people can appropriately use information to guide behavior without awareness.

## INTRODUCTION

How is it possible to safely drive home, yet have little awareness of the trip? This common experience occurs during driving and walking – people can arrive at their location with little awareness of the trip and no memory for objects passed along the way. In some cases, people may arrive at a typical target location but fail to stop at an intended location – you may find yourself at home having failed to stop at the grocery store on the way as you intended. In other words, people appear able to drive and walk without complete awareness of performing the navigation task.

There are two broad categories of possible explanations. One possible explanation is that people were aware of the road and obstacles during the drive, but immediately forgot those features. Alternatively, driving without awareness may represent a form of inattentional blindness, in which objects that pass through the focal point of vision do not enter awareness (Neisser and Becklen, 1975; Becklen and Cervone, 1983; Mack and Rock, 1998; Simons and Chabris, 1999; Simons, 2000). People may use visual information to guide the control of actions, but may not devote attention to objects. Without attention, people may fail to bind features into objects (Treisman and Gelade, 1980; Wolfe, 2007) and thus may fail to become aware of the things they pass when driving or walking. This second possible explanation would be consistent with the theoretical claim that visual information follows two pathways after low level visual processing (Goodale and Milner, 1992; Jeannerod and Jacob, 2005; Westwood and Goodale, 2011). One pathway is the dorsal pathway which uses visual information to guide action, enabling someone to grab an object or navigate around obstacles. The other pathway is the ventral pathway leading to object recognition and conscious awareness.

Inattentional blindness has been demonstrated in lab studies and naturalistic observations. In lab studies of inattentional blindness, people attend to one aspect of a complex event (counting basketball passes by one of two teams) and fail to notice an unusual event that occurs directly in front of their eyes, such as a gorilla or a woman carrying an umbrella (Neisser and Becklen, 1975; Becklen and Cervone, 1983; Mack and Rock, 1998; Simons and Chabris, 1999). Inattentional blindness also occurs in naturalistic settings caused by cell phone conversations during driving and walking (Strayer et al., 2003; Strayer and Drews, 2007; Hyman et al., 2010). In driving simulators, cell phone use leads to decreased recognition of objects that individuals drove past, even though they were just as likely to have looked at the objects (Strayer et al., 2003). People will fail to notice a unicycling clown when talking on a cell phone while walking (Hyman et al., 2010) and a fight when running and tracking another person (Chabris et al., 2011). Inattentional blindness occurs because divided attention in a complex environment decreases awareness of objects that are not the focus of attention. In each of these instances of inattentional blindness, the objects that people failed to notice were interesting and surprising, but were not directly related to the person’s primary task.

Cell phone use not only disrupts awareness in a divided attention situation, but also impacts the control of behavior. People using a cell phone walk more slowly, weave, and change directions more often than people who are not using their cell phones (Hyman et al., 2010), display less safe behavior crossing a street (Neider et al., 2010; Schwebel et al., 2012; Nasar and Troyer, 2013) and experience difficulties using visual information to guide the control of walking through doorways (Lopresti-Goodman et al., 2012). In driving simulators, people using cell phones also display more difficulties controlling the car and responding to changes in the environment (Strayer et al., 2003, 2006; Rakauskas et al., 2004; Kubuse et al., 2006; Törnros and Bolling, 2006; Horrey and Simons, 2007; Drews et al., 2008; Bellinger et al., 2009). Clearly, cell phone use and divided attention disrupts both awareness of the world (the ventral pathway) and the control of behavior (the dorsal pathway).

Nonetheless, we suspect that divided attention may cause greater disruptions to awareness than control of behavior. Even without complete awareness of objects and the environmental layout, people may be able to use visual information to guide walking: people may move to avoid an object without recognizing what the object is. Several lines of evidence are consistent with this possibility.

One line of evidence consistent with a difference between awareness and behavior control comes from differences in perception of slopes and physical responses to slopes. Proffitt et al. (1995) have found that when people provide verbal estimates of the slope of a hill they tend to overestimate that slope. The size of the overestimating error is related to a variety of factors. People estimate the slope is steeper from the top than from the bottom, after a run, and when wearing a heavy backpack (Proffitt et al., 1995; Bhalla and Proffitt, 1999). Nonetheless when people use their hands to directly match the slope of the hill, they accurately create the correct slope. Proffitt (2013) argued that there is a dissociation between awareness and bodily control, a claim that has been questioned by other researchers (Durgin et al., 2009, 2012; Firestone, 2013).

Similar dissociations between awareness and action have been found with visual illusions. People visually perceive lines as different in length in the Müller-Lyer and circles as different in size in the Ebbinghaus illusion. Even when someone understands these illusions, that person will nonetheless see the lines and circles as different sizes. But in versions of the illusions that allow people to perform actions, they do not consistently display the illusions in their behaviors. For example, people see the illusory length difference in the Müller-Lyer illusion, but nonetheless point accurately and walk the correct distance when blindfolded (Wraga et al., 2000; Bruno et al., 2008). Similarly people will accurately set their grasp to allow them to pick up the center circle in the Ebbinghaus illusion (Aglioti et al., 1995; Lee and van Donkelaar, 2002; Culham et al., 2003) and other illusions (Bruno and Bernardis, 2002). Research on dissociations between action and awareness in visual illusions has been criticized because grasping is influenced by visual illusions in many situations (Franz et al., 2000; Franz, 2001; Bruno and Franz, 2009; Schenk et al., 2011). Thus Bruno and Franz (2009) argued that this line of research does not provide compelling evidence for the two visual systems hypothesis.

A more direct dissociation between action and awareness has recently been reported in a visual search task (Solman et al., 2012). Participants were presented with a pile of different shapes on a computer screen and were asked to move the objects to find a particular one. People frequently moved the target without recognizing it as the target. Thus they used visual information to direct behavior without necessarily becoming consciously aware of which object they moved. Usually participants became aware of the target object directly after missing it, but on a small number of trials they moved the target and did not return to the target for several moves.

Navigating in a complex real world environment may sometimes involve behavior being guided by objects that are not consciously recognized. For example, Yanko and Spalek (2014) found that people sometimes lose awareness while driving in a simulator. When probed, participants acknowledged occasions of mind-wandering; that is thinking about something other than the task of driving (see also He et al., 2011). Mind-wandering was associated with changes in driving including faster speed and slower responses to braking events. Individuals were more likely to drive without awareness on routes they had driven more frequently than on novel routes (Yanko and Spalek, 2013).

Becoming aware of an object is generally assumed to require focused attention. People must allocate some attention to bind a set of features to a location (Treisman and Gelade, 1980). Without attention, objects may nonetheless influence a person and guide behavior. If attention is more important for object recognition than directing behavior, then divided attention should be more disruptive of awareness than accurate navigation. Thus cell phone use while walking leads to inattentional blindness for interesting objects near an individual’s path (Hyman et al., 2010). Other forms of distraction and divided attention also lead to inattentional blindness (Chabris et al., 2011) and to mistaken judgments of walking distance (Sargent et al., 2013). In spite of lapses of awareness in these studies, people successfully navigated through complex environments.

In the standard demonstrations of inattentional blindness, people fail to become aware of objects unrelated to their current task. We were interested in something more directly related to the phenomenon of driving and walking without awareness: can people experience inattentional blindness for obstacles that nonetheless guided behavior? We conducted two studies in which we placed obstacles directly in the pathway of walkers and checked if they avoided the obstacles and if they became aware of the obstacles. In essence, our argument is that visual information can be used to guide behaviors but that object recognition is processed separately and is dependent on attention (Treisman and Gelade, 1980; Goodale and Milner, 1992; Jeannerod and Jacob, 2005; Wolfe, 2007; Westwood and Goodale, 2011). Since cell phones may use attentional resources needed for object recognition, we looked at people walking with and without using cell phones. We predicted that even without using cell phones people would sometimes fail to become aware of the obstacles they avoid. In part this should occur because we placed our obstacles in a familiar pathway and this is a situation that should lead to mind-wandering and reduced awareness (Yanko and Spalek, 2013). We also predicted that individuals using their cell phones would be less likely to become aware of the obstacle because this should disrupt the use of focused attention needed for object recognition (Strayer et al., 2003; Hyman et al., 2010).

## STUDY 1

### METHODS

#### Participants

We observed individuals passing a signboard on a campus pathway. Observers rotated through three categories of individual walkers: 52 individuals with no electronics in use, 46 individuals listening to personal music players, and 43 individuals talking on a cell phone. If the observer went more than five minutes without being able to observe a person in the next category, the observer skipped to the following category. Observations were collected of 141 individuals (observers classified 75 as female, 62 as male, and 4 unsure; 124 were classified as college-aged, 11 as older, and 6 unsure).

#### Procedure

We placed a signboard on a pathway and observed when people moved to avoid the sign. The sign was placed at a point where people tend to stay near the edge of the path because the path curves to right approximately 30 feet beyond the placement. The signboard stated “Psychology Research in Progress”. Discreet stakes were placed in the planting area beside the pathway at a distance of 5 and 10 feet before the signboard. Using the stakes, the observers noted at what point the walkers moved to avoid the signboard. The observers worked in pairs and were stationed across the pathway, near the entrance of a building. After each walker passed the signboard, the observers approached to ask a few questions. All walkers were approached 15 feet after passing the signboard such that their backs remained to the signboard. The observers first obtained permission to ask the walker a few questions. If the walker agreed, then the observer asked if they had passed any obstacles on the pathway. If the walker believed they had, then they were asked to identify the obstacle. If they did not volunteer the signboard as the obstacle, they were asked if they had passed a signboard and if they knew what was on the signboard (claiming anything about psychological research was counted as correct and no one said either psychology or research without the other term). Thus we collected both a behavioral measure (when they moved to avoid the signboard) and a perceptual awareness measure (awareness of what obstacle was avoided). Observers worked during normal class periods over a two week period when their schedules and weather permitted. We collected data until we obtained at least 40 observations in each condition (based on other similar studies in our lab we anticipated this would provide adequate power, Hyman et al., 2010).

### RESULTS AND DISCUSSION

Cell phone use disrupted both control of behavior and awareness of the obstacle. In our results, we grouped individuals without electronics and those listening to music players. We planned throughout the study to combine these groups because we did not anticipate any differences based on previous research (Strayer and Johnston, 2001; Consiglio et al., 2003; Hyman et al., 2010; Neider et al., 2010; Walker et al., 2012). Preliminary analyses also indicated no differences between individuals with music players and individuals without any electronics.

People walking while talking on their cell phones were more likely to wait until they were within 5 feet of the signboard before changing their path to avoid the signboard than were individuals and people listening to music players [*χ^2^* (1, *N* = 141) = 5.58, *p* = 0.018]. Although most individuals moved early to avoid the signboard, 25.82% of the cell phone users waited until they were within five feet whereas only 10.20% of non-cell phone users waited until within five feet. This finding is consistent with other research findings showing that cell phone users display difficulty with behavioral control when walking (Consiglio et al., 2003; Nasar et al., 2008; Bellinger et al., 2009; Hyman et al., 2010) and when driving in a simulator (Strayer and Johnston, 2001; Strayer et al., 2003). **Table 1** presents the outcome measures grouped by cell phone users, music player users, and individuals without electronics. This provides additional information showing that cell phone users typically perform differently than other walkers.

**Table 1:** Measures of behavior and awareness based on cell phone use in Study 1: the signboard.

	Walking condition		
	Cell phone	Music player	No electronics
Moved within 5 feet	25.8% (11/43)	10.9% (5/46)	9.6% (5/52)
Answered questions	62.8% (27/43)	95.7% (44/46)	88.5% (46/52)
Saw signboard	63.0% (17/27)	77.3% (34/44)	89.1% (41/46)
Knew content	55.6% (15/27)	72.7% (32/44)	82.6% (38/46)

When approached by the researchers, cell phone users were less likely to agree to respond to questions than were other walkers [*χ^2^* (1, *N* = 141) = 18.14, *p*< 0.001]. Only 62.79% of cell phone users agreed to respond whereas 91.84% of other walkers agree to participate. This may, of course, limit the accuracy of the awareness data for cell phone users. Most likely the cell phone users who refused to answer questions were those most engaged in their phone conversations. This would imply that they were less aware of their environment since cell phone conversations lead to inattentional blindness. In other words, while losing cell phone users was a problem, we may have lost individuals less aware of their surroundings, working against the pattern of the findings.

We then checked if the walkers were aware that they had walked past a signboard. Consistent with inattentional blindness, cell phone users were less likely to be aware that they had passed a signboard [*χ^2^* (1, *N* = 117) = 5.13, *p* = 0.024]. While 83.33% of individuals without electronics and individuals listening to music were aware that they had passed a signboard, only 62.96% of cell phone users were aware. When asked if they knew what was on the signboard, the difference between cell phone users (55.56%) and other walkers remained [77.78%; *χ^2^* (1, *N* = 117) = 5.16, *p* = 0.023].

We next investigated whether when people moved was related to the awareness of the obstacle. We did not have a clear set of predictions here. One possibility is that moving late (within 5 feet of the signboard) would reflect a lack of awareness of one’s surroundings. We might expect people who moved late to display less awareness; that is more inattentional blindness. On the other hand, people who moved late may have suddenly become aware of the signboard and changed their walking direction in response to this last minute awareness. Thus late movers may have been more aware than early movers. Overall people who waited to move were less likely to be aware of the signboard [*χ^2^* (1, *N* = 117) = 4.65, *p* = 0.031]. For people who moved early, 82.00% were aware of the signboard but only 58.82% of people who moved within 5 feet were aware of the signboard.

As we have already noted, when people moved was related to cell phone use. Therefore we conducted this analysis separately for cell phone users and other individuals. For cell phone users, moving early or late was unrelated to awareness of the signboard [*χ^2^* (1, *N* = 27) = 0.001, *p* = 0.974]. No matter when they moved, only 63% of cell phone users were aware of the signboard. For non-cell phone walkers, people who moved within 5 feet (55.56%) were less aware of the signboard than people who moved before 5 feet [86.42%; *χ^2^* (1, *N* = 90) = 5.56, *p* = 0.018].

Both control of walking and awareness of obstacles were influenced by cell phone use. Cell phone users moved to avoid an obstacle later and were less aware of the obstacle a few moments later than were other walkers. Importantly, people did not walk into the signboard. But for many individuals avoiding the obstacle did not lead to awareness of the obstacle. This is a real world demonstration of walking without awareness. The observation that people who moved to avoid the obstacle at the last moment were actually *less* likely to be aware of the object is important for this phenomenon. To some extent, we might have anticipated these individuals would become suddenly aware as they noticed and responded within 5 feet. Instead the visual information was sufficient to guide behavior without leading to conscious awareness. This is possibly a demonstration of a dissociation of behavior control and awareness. Such a dissociation is consistent with the claim that there are two pathways for visual information leading to behavior control and awareness (Goodale and Milner, 1992; Jeannerod and Jacob, 2005; Westwood and Goodale, 2011). This finding is also consistent with other instances in which awareness and body responses are inconsistent and appear somewhat dissociated (Aglioti et al., 1995; Wraga et al., 2000; Bruno and Bernardis, 2002; Proffitt, 2006; Bruno et al., 2008; Solman et al., 2012). Nonetheless, it is possible that participants were aware of the signboard but quickly forgot the object as they moved past the object. This interpretation would be consistent with criticisms of the two visual systems hypothesis (e.g., Bruno and Franz, 2009). For this reason, in our second study we used an unusual stimulus that we expected would result in distinct behaviors if walkers became aware of the stimulus – money on a tree.

## STUDY 2

This study was inspired by “The Money Tree,” a YouTube video in which Rosenthal (2010) placed 100 one-dollar bills on a tree. Although she was interested in watching the excited responses as people discovered the money, she observed that people generally failed to become aware of the money, even after avoiding the tree while walking and often after looking directly at the tree. With her permission, we examined her original 1 h recording from which the YouTube video was edited. Consistent with her claim, we found that few people became aware of the money. We judged awareness as stopping to examine or take the money. We recreated the money tree as an observational study.

### METHODS

#### Participants

On several narrow pathways, we observed people as they passed money hanging on a branch over the path. Observations were collected of each individual who passed the money tree. If someone else was stopped to examine the money as an individual went by, we did not collect observations of the additional person since social interaction added an additional uncontrolled aspect to the situation. We observed 396 individuals (observers classified 193 as female and 203 as male; 375 were classified as college-aged and 21 as older). Most individuals were not using any electronic devices (268 individuals), 65 were using music players, 33 were talking on their cell phones, and 30 were texting.

#### Procedure

**FIGURE 1:**
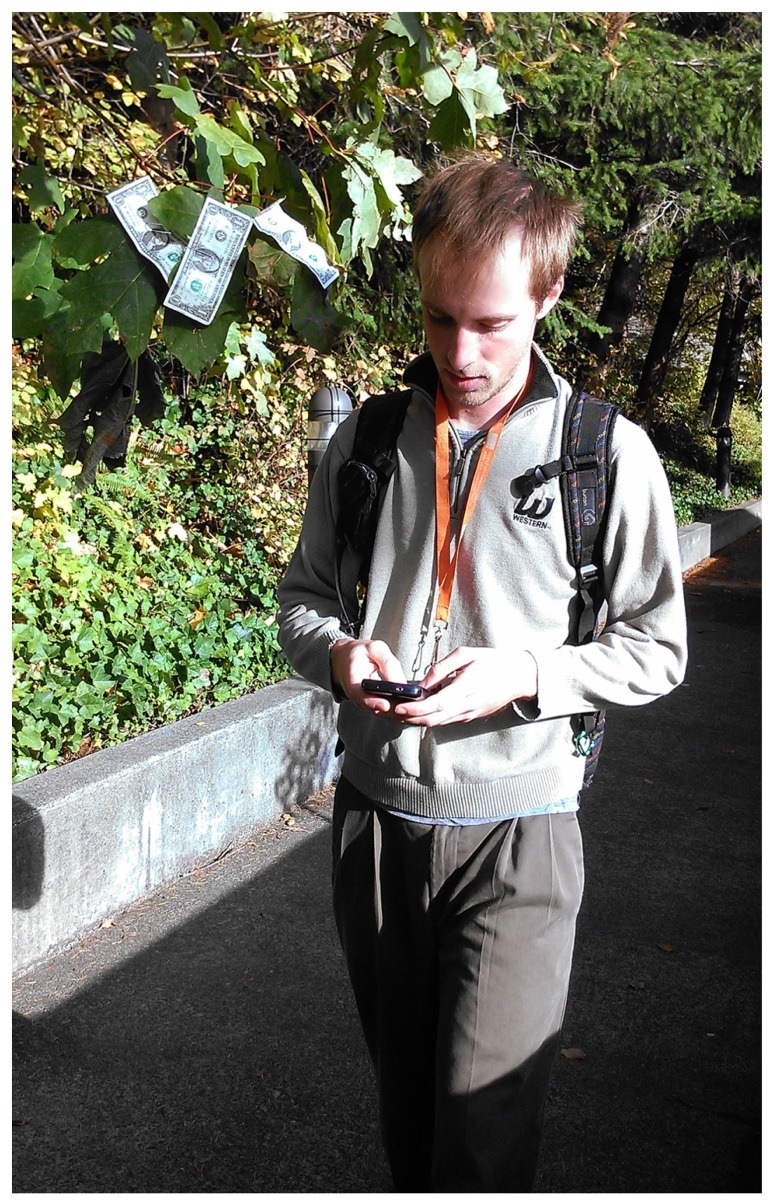
**A research assistant walking past the money tree while texting**.

Three-dollar bills were clipped onto a branch of a deciduous tree beside a narrow path. We used paths between a set of dorms and the academic center of campus. The branch of the tree with the money was bent so that it hung over the path at head height (see **Figure 1** for a photograph of a research assistant walking past the money tree). Since the branch was positioned to extend down over the path, all individuals had to move their heads in order to not walk into the branch. Observers were positioned in pairs about 15 feet beyond the money tree in apparent conversation. Observers collected data over a two-week period as weather and schedules permitted. Observations were collected until at least 30 people were observed in each category.

Because inattentional blindness can result in a failure to become aware of objects that pass directly through the focal point of vision, we depended on behavioral indexes of awareness. We counted individuals as displaying awareness of the money if they stopped to examine the money or if they took the money. Each dollar bill had a message taped to it noting that this was part of a psychology research project. Thus some people examined but did not take the money.

### RESULTS AND DISCUSSION

As with Study 1, we planned to combine walkers without electronics with walkers with music players because previous research has found no differences between these conditions. We first compared and found no difference between cell phone talkers and texters. Thus we collapsed across these conditions. Individuals using their cell phones to talk or text were less likely to display clear evidence of awareness of the money (6.35%; 4 of 63) than those with no electronics or with music players (19.82%; 66 of 333). Thus cell phone use disrupted awareness of objects that people avoided while walking [*χ^2^* (1, *N* = 396) = 6.61, *p* = 0.010]. Although people rarely displayed overt awareness of the money, only 12 people (3.0%) walked into the branch with the money. Given so few observations, it was impossible to discern any difference based on walking condition for walking into the tree. We did not, however, observe any individual who walked into the tree stopping to take the money. In this fashion we observed that people can walk past potentially interesting objects and fail to display overt awareness of the objects. Most individuals failed to become aware of money on a tree.

We combined people talking and texting with a cell phone because we found no difference between these conditions – any use of a cell phone disrupted awareness of the money. From a working memory perspective (e.g., Baddeley and Hitch, 1974), texting should have been more disruptive because both texting and recognizing objects depend on the visual-spatial sketchpad aspect of working memory. We may have failed to find such an effect because of floor effects – almost no one with a cell phone displayed awareness of the money. Another possible explanation is that recognizing an object depends on executive control and any cell phone use also depends on executive control. At this point we cannot be sure if there is no difference between texting and talking or if we simply were unable to observe the difference in this study.

## DISCUSSION

In two studies we observed that people can avoid obstacles in the walking path but nonetheless display little immediate awareness for what the object is and no memory for the object within a few moments of passing. Cell phone users were more likely to display this lack of awareness indicating the importance of attention for becoming aware of and recognizing objects. Of course many people listening to music and individuals who were not using any electronic devices also failed to remember passing a signboard and did not display awareness of money hanging on a tree. Failure to become aware of one’s surroundings in these instances may represent an instance of mind-wandering while walking. The people may have become focused on their own thoughts and been less aware of their surroundings (He et al., 2011; Yanko and Spalek, 2013, 2014).

These naturalistic observations may be demonstrations of how people can drive home and seemingly have little awareness while driving and no memory for the trip after arriving home. People can use information about an object to guide behavior without becoming aware of what the object is – a clear dissociation between the guidance of behavior and awareness. These studies provide the evidence that people can experience inattentional blindness for objects that guided behavior. This is an important extension of inattentional blindness studies, since both traditional lab studies (Neisser and Becklen, 1975; Becklen and Cervone, 1983; Mack and Rock, 1998; Simons and Chabris, 1999) and naturalistic studies (Hyman et al., 2010; Chabris et al., 2011) have only demonstrated awareness failures for objects unrelated to the ongoing task. In another similar demonstration, Solman et al. (2012) recently found that people can move an object during a visual search task and not recognize that the object moved is the one for which they were searching.

One possible explanation of these findings is that perception may be processed in two somewhat independent pathways: the ventral pathway leading to object recognition and the dorsal pathway guiding behavior (Goodale and Milner, 1992; Jeannerod and Jacob, 2005; Westwood and Goodale, 2011). To the extent that the two pathways are somewhat independent, there should be observable dissociations between awareness and the control of behavior. Several lines of research have found dissociations that are consistent with the two visual pathways hypothesis. People differ in their perception of and behavior responses to the slopes of hills (Proffitt et al., 1995; Bhalla and Proffitt, 1999; Proffitt, 2006). In addition, even when people continue to be aware of visual illusions, the control of their walking and grasping indicates accurate control to match the real rather than perceived size of objects (Aglioti et al., 1995; Wraga et al., 2000; Bruno and Bernardis, 2002; Bruno et al., 2008). Our studies provide naturalistic observations of dissociations between awareness and the guidance of behavior that are consistent with the two visual pathways hypothesis. Particularly interesting for the two pathways argument is that even when people moved to avoid the signboard at the last moment, this did not lead to an increased awareness of the signboard.

The two visual systems hypothesis remains controversial. Bruno and Franz (2009) suggested several possible versions of the hypothesis varying in terms of the extent to which the systems are independent. Our data do not unequivocally support any particular two pathway perspective. Instead our findings demonstrated that people can avoid objects without complete awareness (since they failed to respond to the money) and without awareness a few moments later (since they were unaware they had avoided a signboard). We suspect that attention is more important for object recognition and awareness than for the control of behavior. To recognize and become aware of objects, people must use attention to bind features to locations creating object files (Treisman and Gelade, 1980; Wolfe, 2007). People may have been aware that there was an object, but without attention may not have become aware of what the object was.

Although there is evidence that the dorsal and ventral visual pathways lead to dissociations between awareness and the guidance of movement, clearly the two systems interact in meaningful ways (Bruno and Franz, 2009; Schenk and McIntosh, 2010). We found that while awareness was particularly disrupted by cell phone divided attention, divided attention also impacted the control of behavior. People talking on their cell phones moved later to avoid the obstacle in their pathway than other walkers. This disruption of the guidance of behavior is consistent with other findings concerning the impact of cell phones on both walking behavior (Hyman et al., 2010; Neider et al., 2010; Lopresti-Goodman et al., 2012; Schwebel et al., 2012; Nasar and Troyer, 2013) and driving (Strayer et al., 2003, 2006; Rakauskas et al., 2004; Kubuse et al., 2006; Törnros and Bolling, 2006; Horrey and Simons, 2007; Drews et al., 2008; Bellinger et al., 2009). People may be able to walk and drive with little conscious awareness, but they are not nearly as safe and competent as when awareness is also involved. Divided attention makes people slower to respond to objects. This would suggest that awareness may be necessary to plan for movements further in advance (Bruno and Franz, 2009). Additionally, object recognition is important for making the appropriate response. For example, a driver needs to respond differently to a large truck, a car, a bicyclist, and a pedestrian. Thus awareness appears to be important for guiding behavior – we should not rely on the perceptual auto-pilot to get us safely home. These findings are important since people continue to report wide acceptance of cell phone use during driving and many other activities (Forgays et al., 2014). Reducing cognitive distractions, such as cell phone use, should lead to both more awareness of one’s surroundings and better control of behavior.

We observed inattentional blindness for avoided obstacles in both studies. We also observed that people waited longer to respond to the signboard, showing that divided attention disrupts the control of behavior. While the results of these naturalistic observations are consistent with the two visual pathways hypothesis, they do not provide unimpeachable evidence. People on cell phones may be distracted, but they nonetheless avoided both the signboard and the money tree. Divided attention is known to disrupt memory. Thus cell phone use may have disrupted holding the awareness of the obstacles in working memory. This possibility is certainly consistent with the findings of our studies as well. Increased forgetting from working memory is also consistent with the general phenomenon of driving home and realizing one has no awareness of the trip. Conceivably one could have been aware of the drive and the obstacles during the drive. But if working memory was occupied with other concerns, such as a cell phone call or mind-wandering, then the information might have been quickly lost from memory.

Our observations provide empirical examples of people walking, avoiding obstacles, and displaying little awareness of the obstacles. People can pass a signboard and fail to be aware of having done so within a few moments. People can walk past a tree, move to avoid a branch, and fail to become aware of money hanging directly in front of their faces. Apparently people may be able to guide behavior without awareness. Inattentional blindness for objects one avoids is a form of mindless wandering that allows us to walk and drive without awareness of avoided obstacles.

## Conflict of Interest Statement

The authors declare that the research was conducted in the absence of any commercial or financial relationships that could be construed as a potential conflict of interest.
